# Combining Flow and Mass Cytometry in the Search for Biomarkers in Chronic Graft-versus-Host Disease

**DOI:** 10.3389/fimmu.2017.00717

**Published:** 2017-06-19

**Authors:** Arwen Stikvoort, Yang Chen, Emelie Rådestad, Johan Törlén, Tadepally Lakshmikanth, Andreas Björklund, Jaromir Mikes, Adnane Achour, Jens Gertow, Berit Sundberg, Mats Remberger, Mikael Sundin, Jonas Mattsson, Petter Brodin, Michael Uhlin

**Affiliations:** ^1^Department of Oncology-Pathology, Karolinska Institute, Stockholm, Sweden; ^2^Science for Life Laboratory, Department of Medicine, Karolinska Institute, Stockholm, Sweden; ^3^Department of Clinical Sciences, Intervention and Technology (CLINTEC), Karolinska Institute, Stockholm, Sweden; ^4^Centre for Allogeneic Stem Cell Transplantation (CAST), Karolinska University Hospital, Stockholm, Sweden; ^5^Department of Medicine, Karolinska Institute, Stockholm, Sweden; ^6^Department of Infectious Diseases, Karolinska University Hospital, Stockholm, Sweden; ^7^Department of Clinical Immunology and Transfusion Medicine, Karolinska University Hospital, Huddinge, Sweden; ^8^Hematology/Immunology/HSCT Section, Astrid Lindgren Children’s Hospital, Karolinska University Hospital, Stockholm, Sweden; ^9^Department of Neonatology, Karolinska University Hospital, Stockholm, Sweden; ^10^Department of Applied Physics, Royal Institute of Technology, Stockholm, Sweden

**Keywords:** immunophenotyping, hematopoietic stem cell transplantation, graft-versus-host disease, flow cytometry, mass cytometry

## Abstract

Chronic graft-versus-host disease (cGVHD) is a debilitating complication arising in around half of all patients treated with an allogeneic hematopoietic stem cell transplantation. Even though treatment of severe cGVHD has improved during recent years, it remains one of the main causes of morbidity and mortality in affected patients. Biomarkers in blood that could aid in the diagnosis and classification of cGVHD severity are needed for the development of novel treatment strategies that can alleviate symptoms and reduce the need for painful and sometimes complicated tissue biopsies. Methods that comprehensively profile complex biological systems such as the immune system can reveal unanticipated markers when used with the appropriate methods of data analysis. Here, we used mass cytometry, flow cytometry, enzyme-linked immunosorbent assay, and multiplex assays to systematically profile immune cell populations in 68 patients with varying grades of cGVHD. We identified multiple subpopulations across T, B, and NK-cell lineages that distinguished patients with cGVHD from those without cGVHD and which were associated in varying ways with severity of cGVHD. Specifically, initial flow cytometry demonstrated that patients with more severe cGVHD had lower mucosal-associated T cell frequencies, with a concomitant higher level of CD38 expression on T cells. Mass cytometry could identify unique subpopulations specific for cGVHD severity albeit with some seemingly conflicting results. For instance, patients with severe cGVHD had an increased frequency of activated B cells compared to patients with moderate cGVHD while activated B cells were found at a reduced frequency in patients with mild cGVHD compared to patients without cGVHD. Moreover, results indicate it may be possible to validate mass cytometry results with clinically viable, smaller flow cytometry panels. Finally, no differences in levels of blood soluble markers could be identified, with the exception for the semi-soluble combined marker B-cell activating factor/B cell ratio, which was increased in patients with mild cGVHD compared to patients without cGVHD. These findings suggest that interdependencies between such perturbed subpopulations of cells play a role in cGVHD pathogenesis and can serve as future diagnostic and therapeutic targets.

## Introduction

Hematopoietic stem cell transplantation (HSCT) is an established curative treatment for several genetic, metabolic, and hematologic disorders. The idea for cure in HSCT is to replace a recipient’s diseased or impaired immune system with the immune system of a healthy donor (1–3). After the recipient’s immune system is compromised by a conditioning regimen including chemotherapeutic agents and/or irradiation, patients receive a new donor hematopoietic system in the form of hematopoietic stem cells (4). A common complication after HSCT is graft-versus-host disease (GVHD). The pathogenesis of GVHD is caused by an attack of donor immune cells on healthy tissues in the recipient due to incompatibility of major and minor histocompatibility antigens (5–7). By definition, chronic GVHD (cGVHD) develops more than 3 months after HSCT (6). cGVHD can manifest as mild, moderate, or severe (diagnosed and assigned according to the NIH criteria) (8), of which the severe type has a high mortality rate (9).

As of yet, no clear predictive biomarkers have been identified for diagnosis or progression to severe GVHD, though some soluble markers and cellular subsets of interest have been identified. Several studies have identified soluble biomarkers addressed at predicting acute GVHD. One of the first successful studies that attempted to screen for diagnostic plasma biomarkers was able to identify a combination of four proteins (IL-2Rα, TNFR1, IL8, and hepatocyte growth factor) that were predictive of acute GVHD status at time of diagnosis (10). A later study identified a heightened Reg3α level in plasma at diagnosis to be associated with gastrointestinal GVHD onset and severity (11). More recently, a study identified that high levels of plasma ST2 at onset of GVHD therapy could be used as a diagnostic biomarker for increased risk of therapy resistant acute GVHD (12). Another example is a study that assessed the expression of circulating microRNAs and found several specific microRNAs to be diagnostic of acute GVHD and overall survival (13).

Other groups have also focused on combining potential prognostic biomarkers to develop an algorithm to predict risk of acute GVHD development. For example, it was shown that differently combining soluble markers ST2, Reg3α, TNFR1, and IL-2Rα (with the ST2 and Reg3α combination yielding the best results) could stratify patients in high and low risk groups for non-relapse mortality (NRM), response to treatment, and onset of lethal acute GVHD post-HSCT (14, 15). Another study found plasma levels of TIM3, IL6, and sTNFR1 to be predictive of grade III–IV acute GHVD, wherein TIM3 was found to be predictive as soon as day 14 post-HSCT. In this study, ST2 was not correlated to acute GVHD directly, but it was predictive of NRM within 1 year post-HSCT (16). This study corroborates a previous study where they identified TIM3 to be predictive of severe acute GVHD (17). Moreover, a recent study also identified a dual predictive function of ST2 and TIM3 for NRM at 2 years post-HSCT (18). While some difference in importance in biomarkers between papers can be found, it would seem that ST2, TIM3, and TNFR1 can be thought of as promising diagnostic and prognostic soluble biomarkers for acute GVHD.

Many studies have also focused on identifying diagnostic and prognostic biomarkers for cGVHD. Some examples of soluble markers that have been identified are TNF-α (19, 20), sIL-2Rα (21, 22), B-cell activating factor (BAFF) (21–24), sCD13 (21, 22), CXCL9 (18, 22, 25), ST2 (25), IL-15 (26), and soluble MICA (27), to name a few.

In addition, several studies have focused on identifying cellular subsets to utilize as potential biomarkers, be it for acute (28, 29) or cGVHD (20, 24, 30, 31).

However, many questions surrounding cGVHD pathogenesis remain, likely due to a result of the multifaceted nature of cGVHD with different appearances in each of the many putative target organs, but also as a consequence of the immunosuppressive drugs used in these patients.

The process of finding biomarkers is not an easy task and those found must adhere to several guidelines as illustrated in depth by the 2014 report of NIH cGVHD biomarker working group (32). Most importantly, a potential biomarker must be confirmed in at least two independent statistically powerful enough cohorts to be deemed a true potential biomarker. One of the reasons for the difficulty in finding biomarkers is that patients with varying grades of cGVHD receive varying doses of immunosuppression. This difference in immunosuppression makes it harder to compare between cGVHD grades. Another reason is that conventional methods like flow cytometry have a limited number of variables that can be analyzed simultaneously. It is therefore difficult to identify novel cellular subsets in an objective fashion. We took advantage of the increased parameterization offered by mass cytometry to more comprehensively profile these patients in order to find novel immune system alterations and/or putative biomarkers associated with cGVHD (33–35). This technology potentially allows for over 100 markers to be combined simultaneously to characterize individual cells. The benefit beyond the increased number of parameters is the mostly non-overlapping mass tags, circumventing the problem of signal overlap that limits conventional flow cytometry analysis. Indeed, mass cytometry has previously been successfully used to broadly profile immune system variation (36–40). Ultimately, we validated the mass cytometry results by translating and applying the main findings into smaller flow cytometry panels. Since mass cytometry is still a relatively new method, validation by other methods is vital, as is the norm for new techniques (41).

Here, we set out to better understand immune system perturbations in patients with cGVHD. We used high-dimensional mass cytometry-based comparisons between patients with no, mild, moderate and severe cGVHD. We identified clusters of cells across T, B, and NK-cell lineages that were differently regulated between these patient groups. In particular, cells of B and NKT-cell phenotypes distinguished these patient groups, suggesting a particular importance for these populations in cGVHD pathogenesis and severity.

## Materials and Methods

### Patients

All patients were at least 1 year post-HSCT, and none had an ongoing acute GVHD, suspicion of late onset acute GVHD or overlap syndrome. Acute GVHD was diagnosed based on clinical symptoms and/or biopsies according to standard criteria (8). A total of 68 patients with samples from 75 time points were included in the study. Seven patients were sampled at a second time point for the confirmation study by flow cytometry. Four of these seven patients retained the same cGVHD status, while three changed cGVHD severity. The seven patients were never analyzed for both time points in the same experiment. Patients were initially graded for cGVHD at the time of treatment. The grading was retrospectively confirmed, according to NIH scoring for cGVHD, by studying the medical records around the time of blood sample donation; no cGVHD (*n* = 26), mild (*n* = 16), moderate (*n* = 17), and severe cGVHD (*n* = 12) (8). Patient characteristics are displayed in Tables 1 and 2. Table S1 in Supplementary Material clarifies which patient was analyzed for each method. Due to sampling restrictions, it was not possible to analyze all patients for all methods, a selection based on sample availability was made. In addition, it was unfortunately not possible to compare the findings from this study with the immune phenotype of the patients before cGVHD was diagnosed. The study was approved by the Regional Ethical Review Board in Stockholm, Sweden (DNR 2007/1349-31 and DNR 2006/1433-31/3). All patients gave written informed consent in accordance with the amended Declaration of Helsinki.

**Table 1:** The patient characteristics of the 53 patients who were analyzed for the main part of the study (Figures 1–4).

cGVHD	None	Mild	Moderate	Severe
*n*	17	13	12	11
Age [median (range)]				
Patient	50 (34–71)	51 (30–65)	53 (29–63)	39 (15–60)
Donor	29 (22–72)	34 (18–58)	46 (30–63)	35 (17–62)
Gender (male/female)				
Patient	10/7	5/8	8/4	7/4
Donor	11/6	4/9	7/5	5/6
Stem cell source				
BM	0	1	1	2
PBSC	16	11	11	9
BM + PBSC	1	1	0	0
Matching				
Sibling/unrelated/haplo	7/10/0	2/10/1	9/3/0	7/4/0
Diagnosis				
Solid tumor	1	1	0	1
AML	6	9	3	4
ALL	2	2	2	1
MDS/MPS	4	1	2	3
MM	0	0	1	0
CML	0	0	1	0
CLL	1	0	1	0
Lymphoma	3	0	2	2
Prophylaxis				
CsA + MTX	11	10	10	7
Tac + Sir	5	2	2	4
PTCy	1	1	0	0
Conditioning				
RIC/MAC	14/3	10/3	7/5	5/6
Anti T-cell antibody treatment				
Yes/no	10/7	10/3	3/9	5/6
aGVHD				
None/I/II/III	11/4/2/0	7/3/2/1	2/2/6/2	2/2/3/4
Systemic immunosuppressive treatment at inclusion				
Yes/no	2/15	6/7	12/0	10/1

*cGVHD, chronic graft-versus-host disease; n, number; BM, bone marrow; PBSCs, peripheral blood stem cells; AML, acute myeloid leukemia; ALL, acute lymphoblastic leukemia; MDS, myelodysplastic syndrome; MPS, myeloproliferative syndrome; MM, multiple myeloma; CML, chronic myeloid leukemia; CLL, chronic lymphocytic leukemia; CsA, cyclosporine; MTX, methotrexate; Tac, tacrolimus; Sir, sirolimus; PTCy, posttransplant cyclophosphamide; RIC, reduced intensity conditioning; MAC, myeloablative conditioning; aGVHD, acute graft-versus-host disease*.

**Table 2:** The patient characteristics of the 37 patients who were analyzed in the confirmatory flow cytometry study (Figure 5).

cGVHD	None	Mild	Moderate	Severe
*n*	15	7	10	5
Age [median (range)]				
Patient	62 (40–70)	34 (28–65)	54 (29–67)	57 (26–67)
Donor	27 (22–60)	27 (23–58)	41 (23–63)	35 (28–56)
Gender (male/female)				
Patient	11/4	5/2	8/2	3/2
Donor	14/1	4/3	4/6	2/3
Stem cell source				
BM	0	1	2	1
PBSC	15	6	8	4
Matching				
Sibling/unrelated/haplo	1/14/0	1/5/1	8/2/0	3/2/0
Diagnosis				
Solid tumor	0	0	0	1
AML	10	6	3	0
MDS/MPS	4	1	1	3
MM	0	0	2	0
CML	0	0	1	0
CLL	1	0	1	0
Lymphoma	0	0	2	1
Prophylaxis				
CsA + MTX	1	5	8	4
Tac + Sir	3	1	2	1
PTCy	1	1	0	0
Conditioning				
RIC/MAC	14/1	3/4	7/3	4/1
Anti T-cell antibody treatment				
Yes/no	14/1	5/2	2/8	3/2
aGVHD				
None/I/II/III	8/5/2/0	3/3/1/0	2/3/3/2	1/0/2/2
Systemic immunosuppressive treatment at inclusion				
Yes/no	0/15	4/3	10/0	5/0

*cGVHD, chronic graft-versus-host disease; n, number; BM, bone marrow; PBSCs, peripheral blood stem cells; AML, acute myeloid leukemia; ALL, acute lymphoblastic leukemia; MDS, myelodysplastic syndrome; MPS, myeloproliferative syndrome; MM, multiple myeloma; CML, chronic myeloid leukemia; CLL, chronic lymphocytic leukemia; CsA, cyclosporine; MTX, methotrexate; Tac, tacrolimus; Sir, sirolimus; PTCy, posttransplant cyclophosphamide; RIC, reduced intensity conditioning; MAC, myeloablative conditioning; aGVHD, acute graft-versus-host disease*.

### Sample Preparation

Plasma was separated from whole blood samples and stored at −80°C. Peripheral blood mononuclear blood cells (PBMCs) were separated by density gradient centrifugation [800 × *g*, 20 min; Lymphoprep (Fresenius Kabi, Oslo, Norway)] and frozen at −196°C in 10% DMSO in complete RPMI-1640 medium [HyClone^®^ (Thermo Fisher Scientific Inc., Waltham, MA, USA)], enriched with 10% human AB-serum (Karolinska University Hospital, Huddinge, Sweden), 2 mM l-glutamine (Gibco, Life Technologies, Paisley, UK), 100 IU/ml penicillin G (Gibco), and 100 mg/ml streptomycin (Gibco).

### Enzyme-Linked Immunosorbent Assay (ELISA)

B-cell activating factor levels in plasma samples were determined using an ELISA. The test was done according to the manufacturer’s instructions [Human BAFF/Blys/TNFSF13B Quantikine ELISA kit (R&D Systems Inc., Minneapolis, MN, USA)]. A Vmax Kinetic ELISA Microplate Reader (Molecular Devices, LLC, Sunnyvale, CA, USA) was used for the analysis.

### Multiplex Assay

Plasma levels of 26 chemokines and cytokines were determined using the MILLIPLEX MAP Human Cytokine/Chemokine—Premixed 26 Plex from Millipore (Millipore Corporation, Temecula, CA, USA) according to the manufacturer’s protocol and as described before (42–44). Analysis was done with the Luminex IS 2.3 software (Luminex Corp., Austin, TX, USA) on the LABScan100 (One Lambda Inc., Canoga Park, CA, USA).

### Flow Cytometry

Peripheral blood mononuclear blood cell staining was performed as previously described (45, 46). For intracellular staining, the protocol of the BD Cytofix/Cytoperm™ kit (BD Biosciences, San Jose, CA, USA) was used. Acquisition was performed with a BD FACS Aria or a BD FACS Canto using BD FACS Diva 7 software (BD Biosciences). The antibodies used for this study are listed in Table S2 in Supplementary Material.

### Mass Cytometry

Cryopreserved PBMC samples from 40 patients (no cGVHD, *n* = 11; mild cGVHD, *n* = 9; moderate cGVHD, *n* = 10; and severe cGVHD, *n* = 10) were thawed in complete RPMI medium (HyClone^®^) supplemented with fetal bovine serum (FBS), penicillin–streptomycin, and benzonase (Sigma-Aldrich, St. Louis, MO, USA) and rested overnight 37°C in 5% CO_2_. For live-dead cell distinction, cells were stained with 2.5 µM cisplatin (Fluidigm, South San Francisco, CA, USA) in RPMI without FBS, for 5 min at room temperature (RT) and quenched with complete RPMI. Cells were then fixed with 1% formaldehyde in dH_2_O (Polysciences Inc., Warrington, PA, USA) and washed, followed by resuspension in CyFACS buffer (PBS with 0.1% BSA, 0.05% sodium azide, and 2 mM EDTA). Next, cells were incubated for 30 min at 4°C with a cocktail of metal-conjugated antibodies targeting the surface antigens. The cells were then washed with CyFACS buffer, fixated with 1% formaldehyde and permeabilized using an intracellular fixation and permeabilization buffer set (eBiosciences Inc., San Diego, CA, USA) as per the manufacturer’s recommendations. The cells were then stained intracellularly with an antibody cocktail for 60 min at RT. Cells were then washed, fixed in 1% formaldehyde, and stained with DNA intercalator (0.125 µM MaxPar^®^ Intercalator-Ir, Fluidigm). Cells were subsequently washed with CyFACS buffer, PBS, and milliQ water, filtered through a 35 µm nylon mesh, diluted to 500,000 cells/ml, and acquired at a rate of 300–500 cells/s using a CyTOF2 (Fluidigm) mass cytometer, CyTOF software version 6.0.626 with noise reduction, a lower convolution threshold of 200, event length limits of 10–150 pushes, a sigma value of 3, and flow rate of 0.045 ml/min.

Purified antibodies were obtained in carrier/protein-free buffer and then coupled to lanthanide metals using the MaxPar antibody conjugation kit (Fluidigm) as per the protocol obtained from the manufacturer. After determining the protein concentration by measurement of absorbance at 280 nm, the metal-labeled antibodies were diluted in CANDOR PBS Antibody Stabilization solution (CANDOR Bioscience, Wangen, Germany) for long-term storage at 4°C. The mass cytometry antibody markers, sources, and metal tags used for this study are listed in Table S3 in Supplementary Material.

### Data Analysis

Flow cytometry data analysis was done with FlowJo software (Tree Star, Inc., Ashland, OR, USA). Fluorescence-minus-one samples were used to obtain proper gating strategies (47). Mass cytometry data were analyzed with Citrus software (48) and ACCENSE software (49) (http://www.cellaccense.com). Small adjustments to the Citrus software code were made to allow the export of single-cell data for calculation of cluster sizes and plotting within tSNE-maps for ACCENSE.

Univariate statistical analysis was done with the Kruskal–Wallis test (KW), Mann–Whitney *U* test (MW), Pearson’s χ^2^ test (χ^2^), and Fisher’s exact test (FE) using IBM SPSS Statistics 23 (IBM, Armonk, NY, USA) software. Where appropriate, the Bonferroni correction was used in *post hoc* analysis. Statistical significance was set at *p* < 0.05, two-tailed. The Citrus software performed the statistical analysis of the mass cytometry data automatically.

Data in tables are presented as median values and range (minimum–maximum) or as absolute numbers. In graphs, data are shown as concentrations or frequency of cells from indicated cell subsets. Graphs were made using Prism 6 (GraphPad, San Diego, CA, USA) software.

## Results

### Patient Characteristics

Four patient groups were selected based on cGVHD status; none, mild, moderate, and severe; according to the NIH criteria (8). The patient groups were similar in terms of age, gender, stem cell source, donor type, diagnosis, conditioning regimen, and prophylactic treatment (KW, Table 1). Anti-T-cell antibodies were given to approximately half of all patients during the preconditioning regimen. However, patients without cGVHD or mild cGVHD received anti-T-cell antibodies more often than patients who developed moderate or severe cGHVD (χ^2^, no versus mild versus moderate versus severe cGVHD, *p* = 0.065; FE, no and mild versus moderate and severe cGVHD, *p* = 0.028). In addition, severity of acute GVHD was linked to severity of cGVHD (χ^2^, *p* = 0.035). There was no difference in the engraftment time of leukocytes, neutrophils, or thrombocytes. Total leukocyte, thrombocyte, and granulocyte counts were similar at time of inclusion.

### Immunosuppression

Due to the severity of cGVHD, patients with moderate or severe cGVHD received high doses of systemic immunosuppressive treatment at the time of inclusion in the study, in contrast to patients without cGVHD and most patients with mild cGVHD (χ^2^, *p* < 0.001). Some patients with mild cGVHD received a low dose of systemic immunosuppressant drugs at the time of inclusion. To take these treatment differences into account, all analyses on blood samples taken at inclusion time were done comparing patients without cGVHD to those with mild cGVHD, and patients with moderate cGVHD to those with severe cGVHD.

### Serum Protein Phenotype

**Figure 1:**
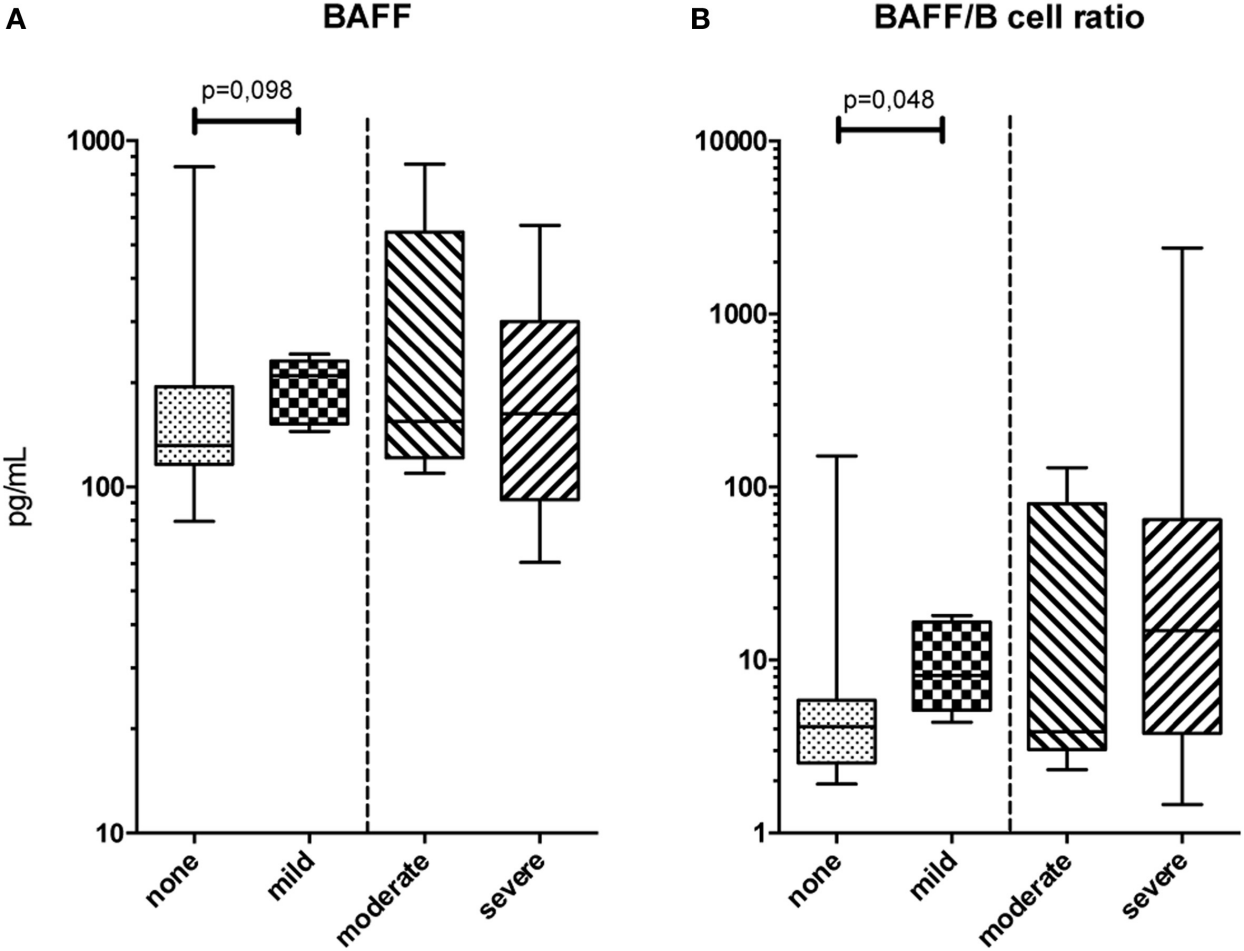
Serum protein phenotype. **(A)** B-cell activating factor (BAFF) levels in peripheral blood and **(B)** BAFF/B-cell ratios for the four chronic graft-versus-host disease (cGVHD) patient groups. Statistical analysis was done with the Mann–Whitney *U* test. *n* = 11, no cGVHD; *n* = 6, mild cGVHD; *n* = 5, moderate cGVHD; and *n* = 8, severe cGVHD.

An extensive soluble phenotype mapping by means of ELISA and multiplex assay was performed. BAFF levels were not found to differ between patient groups (Figure 1A); however, BAFF/B-cell ratios were increased in patients with mild cGVHD compared to patients without cGVHD (MW, *p* = 0.048; Figure 1B). Due to small sample size, it was not possible to analyze the effect of immunosuppressive treatment on BAFF levels within the cGVHD groups. There were no differences in these serum proteins between moderate and severe cGVHD patients. Nor were there any differences in cytokine levels as measured by multiplex assay between the patient groups.

### Conventional Flow Cytometry Immune Cell Phenotype

**Figure 2:**
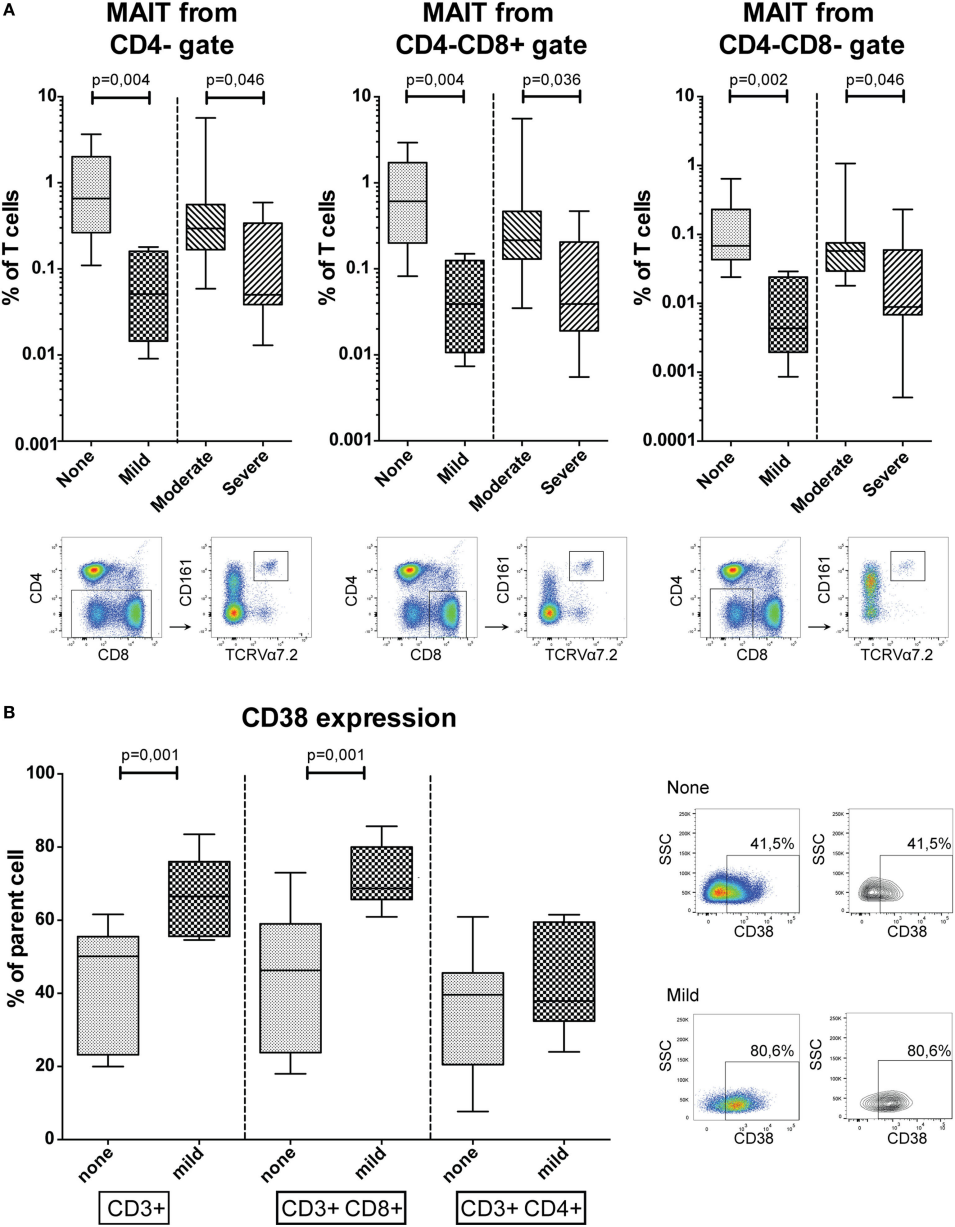
Conventional flow cytometry results. **(A)** The percentage of mucosal-associated T (MAIT)-cells in peripheral blood, defined as CD161+ TCRVα7.2+ T-cells, in CD4−, CD4− CD8+ and CD4− CD8− gates. Representative flow cytometry figures of a patient with severe chronic graft-versus-host disease (cGVHD) are shown below the graphs for each gating strategy. Statistical analysis was done with the Mann–Whitney *U* test (MW). *n* = 9, no cGVHD; *n* = 5, mild cGVHD; *n* = 8, moderate cGVHD; *n* = 9, severe cGVHD. **(B)** CD38 expression in CD3+, CD3+ CD8+, and CD3+ CD4+ T-cells. Representative flow cytometry figures of a patient without cGVHD and with mild cGVHD are shown to the right of the graphs gated on T-cells. Statistical analysis was done with the MW. *n* = 11, no cGVHD and *n* = 7, mild cGVHD.

An extensive flow cytometry panel was set up to analyze the patient groups for a variety of well-defined and distinct T, B, and NK-cell subsets. We observed lower frequencies of blood mucosal-associated T (MAIT) cells, defined as CD161+ TCRVα7.2+ T-cells, in patients with more severe cGVHD (Figure 2A). MAIT-cells gated from CD4− T-cells were reduced in frequency in mild cGVHD patients compared to patients without cGVHD (MW, *p* = 0.004), and in patients with severe cGVHD compared to moderate cGVHD (MW, *p* = 0.046). Similarly, MAIT-cells gated from CD4− CD8+ and CD4− CD8− T-cells were present in lower frequency in mild cGVHD patients (MW, *p* = 0.004 and *p* = 0.002) and in severe cGVHD patients (MW, *p* = 0.036 and *p* = 0.046).

The activation marker CD38 was expressed by a higher percentage of total T-cells (MW, *p* = 0.001) and CD8+ T-cells (MW, *p* = 0.001) among patients with mild cGVHD compared to patients without cGVHD (Figure 2B). This difference was not observed in the CD4+ T-cell population.

No differences were found between the patient groups for other canonical populations such as total T-cells, CD4+ T-cells, B-cells, NK-cells, or memory differentiation populations.

### High-Dimensional CyTOF Immune Cell Analysis

To more thoroughly characterize immune cell populations associated with cGVHD, we used a 33-parameter mass cytometry panel focused on markers expressed on lymphocytes. We analyzed 11 patients without cGVHD, 9 with mild cGVHD, 10 with moderate cGVHD, and 10 with severe cGVHD and searched for high-dimensional cell phenotypes distinguishing these groups.

#### No versus Mild cGVHD

**Figure 3:**
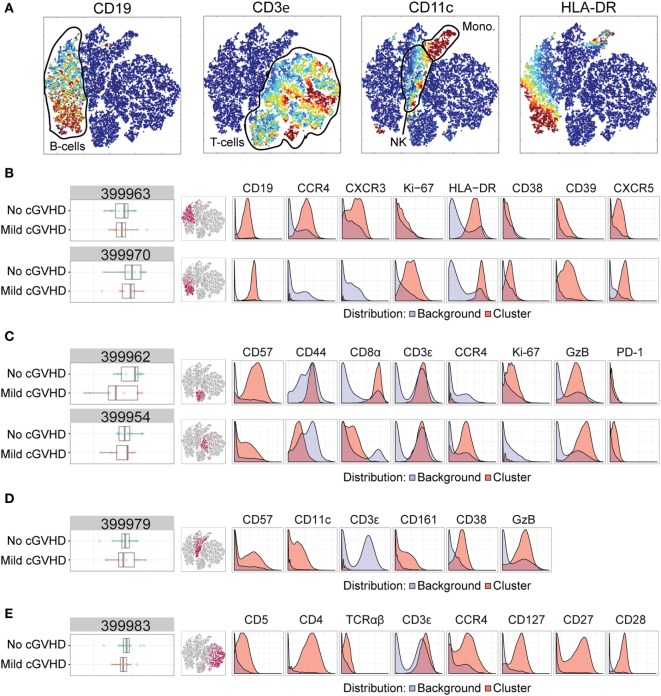
Mass cytometry analysis in patients without chronic graft-versus-host disease (cGVHD) versus patients with mild cGVHD. Results after automated cell clustering software Citrus and ACCENSE (*n* = 11, no cGVHD and *n* = 9, mild cGVHD). The statistical analysis was performed by the Citrus software. The boxplots indicate the spread of the abundancy of the separate clusters, and the histograms depict the expression of specific cellular markers (blue depicts background expression, and red indicates expression of the cluster). **(A)** Multidimensional depiction of some main cellular subsets. **(B)** Two B-cell subsets. Cluster 399963 expressed CD19, HLA-DR, CD39, CXCR5, CCR4, and CXCR3. Cluster 399970 expressed CD19, HLA-DR, CD39, CXCR5, and Ki-67. **(C)** Two NKT-cell subsets. Cluster 399962 expressed CD3, Granzyme B (GzB), CD57, CD44, Ki-67, and CD8. Cluster 399954 expressed CD3, GzB, CD57, CCR4, PD-1, and to a lesser degree CD8. **(D)** An NK-cell subset, cluster 399979, expressed CD57, GzB, CD39, CD11c, and CD161. **(E)** A CD4+ T-cell subset, cluster 399983, expressed CD3, TCRαβ, CD4, CD5, CCR4, CD127, CD27, and CD28.

First, we compared HSCT patients without cGVHD to those with mild cGVHD. We performed standard normalization to internal bead standards, gated on DNA-containing cells and applied the Citrus algorithm for high-dimensional clustering and modeling of differentially regulated features (48). With this algorithm, cells across all samples are merged, hierarchically clustered and subsequently split apart. The algorithm then selects the clusters best distinguishing patients with mild cGVHD from patients without cGVHD, using a nearest shrunken centroid predictive model (48). Figure 3A depicts a multidimensional interpretation of the major immune subsets (T, NK, and B-cells, monocytes) after mass cytometry.

We identified six clusters of interest with differences between patients without cGVHD to patients with mild cGVHD (Figures 3B–E). Two of these (cluster 399963 and 399970) we interpreted as B-cell populations by their expression of CD19, HLA-DR, and CXCR5. Cluster 399963 was additionally characterized by a positive CD39, CCR4, and CXCR3 expression, and lack of the proliferation marker Ki-67. The B-cells in cluster 399970 did not express CCR4 or CXCR3 but were uniformly positive for Ki-67 and CD39. Both of these B-cell subsets were more abundant in patients without cGVHD than in patients with mild cGVHD (Figure 3B).

Two other clusters (399962 and 399954) were interpreted as likely NKT-cells. They expressed CD3, Granzyme B (GzB), and CD57, although cluster 399954 expressed CD57 to a lesser extent than cluster 399962. Furthermore, cluster 399962 expressed CD44, Ki-67, and CD8 to a higher degree than cluster 399954. In contrast, CCR4 and PD-1 were expressed by the cells in cluster 399954 and not by the cells in cluster 399962. Interestingly, cluster 3999962 was more abundant in patients without cGVHD, while cluster 3999954 was more abundant in patients with mild cGVHD suggesting that these shifts might represent NKT-cell phenotypic alterations associated with cGVHD (Figure 3C).

The fifth cluster to be identified (cluster 399979) was more abundant in patients without cGVHD and contained mature NK-cells (negative for CD3, but expressing CD57, GzB, CD39, CD11c, and CD161; Figure 3D). Finally, cluster 399983 was a CD4+ T-cell subset (CD3+, TCRαβ+, and CD4+) expressing CD5, CCR4, CD127, CD27, and CD28. This population was more abundant in patients without cGVHD than in patients with mild cGVHD (Figure 3E).

#### Moderate versus Severe cGVHD

**Figure 4:**
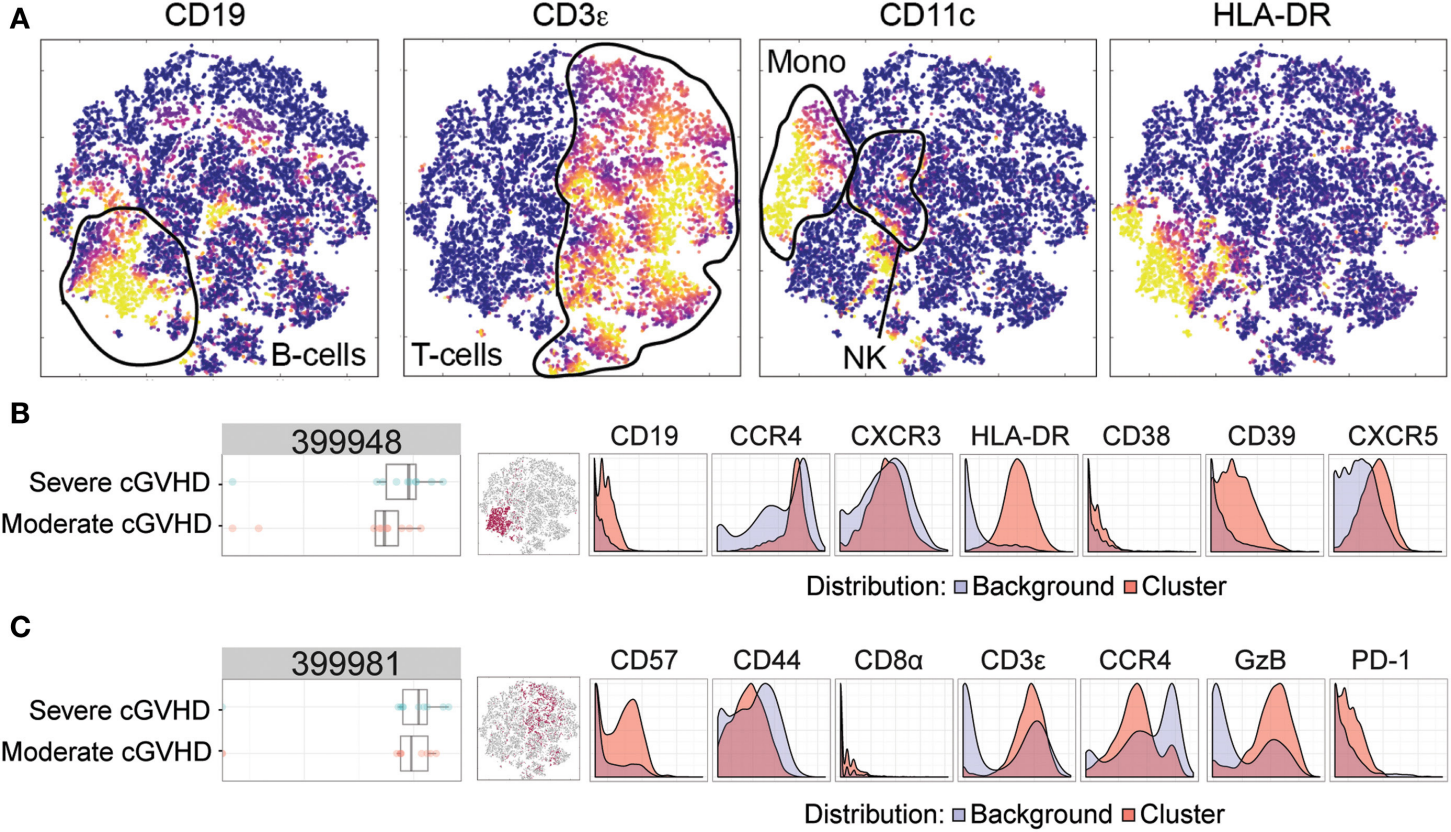
Mass cytometry analysis in patients with moderate chronic graft-versus-host disease (cGVHD) versus patients with severe cGVHD. Results after automated cell clustering software Citrus and ACCENSE (*n* = 10, moderate cGVHD and *n* = 10, severe cGVHD). The statistical analysis was performed by the Citrus software. The boxplots indicate the spread of the abundancy of the separate clusters, and the histograms depict the expression of specific cellular markers (blue depicts background expression, and red indicates expression of the cluster). **(A)** Multidimensional depiction of some main cellular subsets. **(B)** A B-cell subset, cluster 399948, expressed CD19, HLA-DR, CD39, CXCR5, CCR4, CXCR3, and to a slight degree CD38. **(C)** An NKT-cell subset, cluster 399981, expressed CD3, CD57, Granzyme B (GzB), and dimly expressed CD44, PD-1, CD8, and CCR4.

To investigate possible phenotypic alterations associated with an increased severity of cGVHD, we similarly compared patients with moderate cGVHD and severe cGVHD. Figure 4A depicts a multidimensional interpretation of the major immune subsets (T-cells, B-cells, and monocytes) after mass cytometry. Using the same Citrus algorithm, we identified two clusters that could distinguish these patient groups. The first cluster (399948) characterized patients with severe cGVHD from patients with moderate cGVHD (Figure 4B). This B-cell population (CD19+, HLA-DR+) expressed CXCR5, CD39, CCR4, CXCR3, and to a slight degree CD38 (Figure 4B). The second cluster (399981) was also positively correlated with cGVHD severity and was another likely NKT-cell subset with a high expression of CD3, CD57, and GzB, and dim expression of CD44, PD-1, CD8, and CCR4 (Figure 4C).

### Confirmatory Flow Cytometry of Identified Cell Populations

To verify whether high-dimensional phenotypes could also be identified using a reduced 9-parameter flow cytometry panel available in routine clinical practice, we analyzed a cohort of 37 patients. Patient characteristics of this confirmation study are displayed in Table 2. Of these 37 patients, 15 were new to the study and 22 had been included previously (Table S1 in Supplementary Material). New patients and time points were drafted for the confirmation study due to limited sample sizes obtained from the original patients.

**Figure 5:**
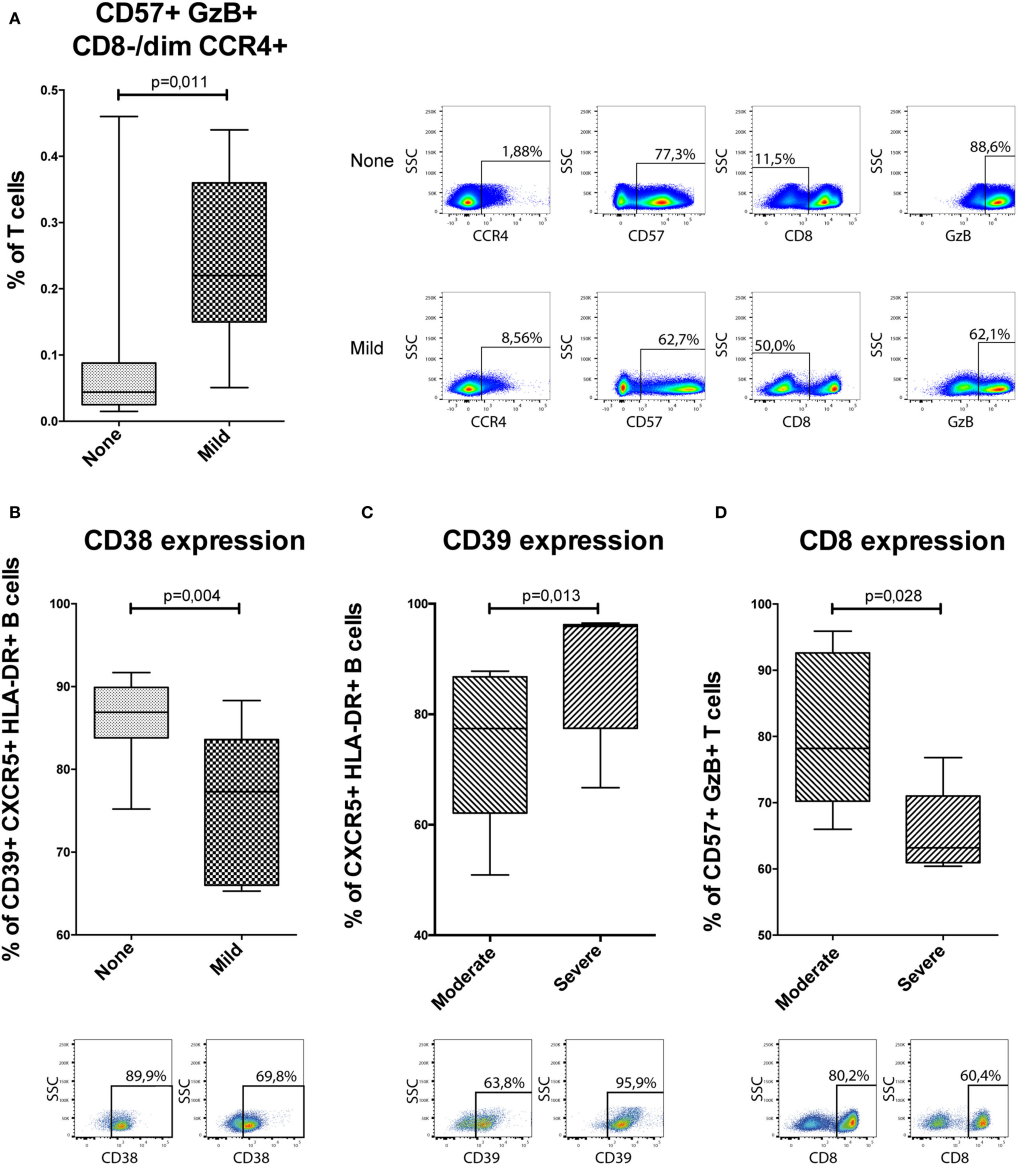
Confirmatory flow cytometry results. Analyzed flow cytometry results for a selection of markers for 37 patients [*n* = 15, no chronic graft-versus-host disease (cGVHD); *n* = 7, mild cGVHD; *n* = 10, moderate cGVHD; and *n* = 5, severe cGVHD]. GzB, Granzyme B. Statistical analysis was done with the Mann–Whitney *U* test. **(A)** A potential NKT-cell subset, based on cluster 399954 (Figure 3C). Representative flow cytometry figures of a patient without cGVHD and with mild cGVHD are shown to the right of the graphs gated on T-cells. **(B)** CD38 expression on CD39+ CXCR5+ HLA-DR+ B-cells. Representative flow cytometry figures of a patient without cGVHD and with mild cGVHD are shown below the graphs. **(C)** CD39 expression on CXCR5+ HLA-DR+ B-cells. Representative flow cytometry figures of a patient with moderate and severe cGVHD are shown below the graphs. **(D)** CD8 expression on CD57+ GzB+ T-cells. Representative flow cytometry figures of a patient with moderate and severe cGVHD are shown below the graphs.

Based on the mass cytometry results (Figures 3 and 4; Figure S1 in Supplementary Material), four flow cytometry panels were designed to identify the clusters (Table S4 in Supplementary Material). Initially, Boolean gating of all involved markers was used to obtain abundancies for each cluster. All eight clusters could be identified in this manner in flow cytometry (Figure 5A; Figure S2 in Supplementary Material). One of the NKT-cell subsets identified in the analysis between patients without GVHD and mild cGVHD (cluster 399954, Figure 3C) was found to be different in the custom-made flow cytometry panel (MW, *p* = 0.011; Figure 5A). To identify this subset in flow cytometry, cells were first gated with a viability dye and consequently gated for lymphocytes and a positive expression of CD3, GzB, CD57, CCR4, and a negative or dim expression of CD8. Patients without cGVHD had a median five times lower frequency of this NKT-cell subset than patients with mild cGVHD.

The data were then analyzed according to conventional sequential gating strategy (Figures 5B–D). Patients without cGVHD had higher frequencies of CD38-expressing CD39+ CXCR5+ HLA-DR+ B-cells as compared to patients with mild cGVHD (MW, *p* = 0.004; Figure 5B). In addition, in patients with severe cGVHD, we could observe an increased frequency of CD39-expressing CXCR5+ HLA-DR+ B-cells (MW, *p* = 0.013; Figure 5C) and a reduced frequency of CD8-expressing CD57+ GzB+ T-cells (MW, *p* = 0.028; Figure 5D) as compared to patients with moderate cGVHD.

Half of the clusters identified by mass cytometry could be recreated and identified in smaller flow cytometry panels, either by looking at abundancies through Boolean gating or by analyzing immune phenotype of the clusters by conventional sequential gating.

## Discussion

Identification of reliable diagnostic markers in relatively easily accessible patient material, such as peripheral blood samples, is vital for improved cGVHD diagnosis. Currently, clinically there are no measurable biomarkers in blood for cGVHD diagnosis. Consequently, a reliable diagnosis of cGVHD often requires organ biopsies, given the variable clinical presentation in different tissues and between patients. Discovering new biomarkers by non-invasive techniques from blood samples using methods such as ELISA for protein profiling, or multiplex serum protein assays as well as cell analyses by flow cytometry has proven to be difficult. One likely reason for this is that these methods typically measure only a handful of parameters at a time and at a specific time point, preventing identification of complex signatures consisting of multiple proteins and/or cells in the blood. High-dimensional immunology methods allow for such signatures to be detected as they can measure multiple proteins and cell types simultaneously, which better characterizes the condition of interest (50).

Research into cGVHD development is often hindered by many confounders, such as differences in patient characteristics and treatment. Therefore, we analyzed the four patient groups for potential confounders. The groups were found to be similar for age, gender, stem cell source, donor type, diagnosis, conditioning regimen, prophylactic treatment, engraftment or leukocyte, thrombocyte, and granulocyte counts. Though not significant between the four groups, there was a trend toward significance between anti-T-cell treatment between the groups. However, this was due to differences in patients without cGVHD or mild cGVHD versus patients with moderate or severe cGVHD; and not present when comparing none to mild cGVHD patients and moderate to severe cGVHD patients (Table 1). All comparisons were done between none to mild cGVHD and moderate to severe cGVHD, hence, anti-T cell treatment should not have affected the results. Unsurprisingly, a history of higher grades of acute GVHD was correlated to severity of cGVHD, as has also been reported by several studies previously (51–53).

In this study, using our 33-marker mass cytometry approach combined with an unbiased and high-dimensional clustering analysis, we were able to identify multiple cell populations that distinguished both patients with cGVHD from patients without cGVHD and also identified signatures correlating with disease severity. The results from the mass cytometry analysis could to a certain extent be confirmed using a more conventional flow cytometry approach, an approach that has also been done by other studies (39, 54). The adjusted flow cytometry panel could provide a more direct clinical utility than a mass cytometry panel.

An extensive soluble and cellular phenotype mapping by means of ELISA, multiplex assay, and flow cytometry was performed. Patients with a higher grade of cGVHD were found to have a higher BAFF/B-cell ratio. This ratio is often used in studies on cGVHD biomarkers, with similar results and is thought to reflect B-cell involvement in cGVHD pathogenesis (21–24, 55). MAIT-cell frequencies were decreased in patients with more severe cGVHD, most likely reflecting a migration toward the site of cGVHD-induced inflammation. This confirms a previous study where they correlated GVHD to a decrease of an IL-17 producing CD161+ CCR6+ T-cell subset, which most likely comprises both Th17-cells (CD4+) and MAIT-cells (CD4−) (56). Conflicting results have been published by a study where Th17-cells were increased in patients with cGVHD (30). However, this study was performed before the TCRVα7.2 antibody was available making it hard to compare the results of these studies. Further research into this topic is needed. A longitudinal study, sampling patients at time points of differing cGVHD grades combined with phenotyping the infiltrating lymphocytes in affected organs, to differentiate between MAIT and Th17-cells, could help elucidate this. Finally, activation marker CD38 was expressed on a higher frequency of (CD8+) T-cells in patients with mild cGVHD, indicating an increased activated state of cytotoxic cells. This was not increased in patients with moderate or severe cGVHD probably due to immunosuppressant treatment. A role of CD38 positive cytotoxic cells has previously been observed in acute GVHD development (57). It has also been linked to changes in B-cell frequencies for cGVHD. Specifically, a recent study identified increased frequencies of CD38hi plasmablasts in cGVHD patients (58). However, to our knowledge, CD38 involvement has not been linked to cytotoxic T-cells for cGVHD.

In this study, we also analyzed 40 cGVHD patients by mass cytometry. Several unique subsets could be identified with specialized automated clustering software. Most differences could be observed between the patients without cGVHD and mild cGVHD. This is most likely due to the fact that immunosuppressive medication used in patients with more severe cGVHD flattens differences in blood immune phenotype, as also observed in a previously referenced study on BAFF (23). The cellular subsets identified by mass cytometry expressed markers, which are not often analyzed in a conventional flow cytometric panel. For instance, CXCR3 and CXCR5 are traditionally associated with being involved in the migration of activated T-cells (59–61) and are seldom used to identify B-cell subsets, although they can be expressed on chronic lymphocytic leukemia (CLL) affected B-cells (62). We conclude that cellular signatures exist that distinguish patients with and without cGVHD but also correlate with cGVHD severity.

After mass cytometry, we selected the cellular markers we believed to be most distinctive for each of the identified clusters and created a condensed flow cytometry panel. Boolean gating for cluster abundancies yielded significance for a cluster of an NKT-cell subset expressing CD3, CD57, GzB, CCR4, and dimly expressing CD8. Patients with mild cGVHD had a larger median frequency of this subset than patients without cGVHD, similar to the mass cytometry results (cluster 399954). In contrast, a decreased frequency of NKT-cells has been observed previously in patients with extensive GVHD (63). Unfortunately, since the mass cytometry panel did not include specific NKT-cell markers, nor were we able to isolate the cells and test for CD1d restriction, we cannot conclude beyond doubt that the cells were invariant NKT-cells. Activated cytotoxic T-cells can express CD57 and produce GzB with a reduced expression of CD8 (64). Strikingly, the increase in either NKT-cells or activated cytotoxic T-cells was only observed when patients without cGVHD were compared to patients with mild cGVHD. Patients with moderate and severe cGVHD had similar abundancies of this cellular subset after flow cytometry. It is possible that the large doses of immunosuppressive drugs these patients received smoothed out differences in immune phenotype between them as has been observed for BAFF concentration (23).

The remaining seven clusters could be identified by Boolean gating in flow cytometry but were not found to be different between patient groups. There are several possible reasons for this. One main cause could be the relatively small cohort size of some of the patient groups. Another reason could be that Boolean gating is not a robust method to identify rare populations, which could explain the lack of significance when comparing abundancies. Hence, the data were also analyzed using the more conventionally used sequential gating strategy for flow cytometry.

Sequential gating allowed for the identification of differences in expression of cellular markers in three clusters. In the panel looking at cluster 399970, an increased frequency of CD38+ CD39+ CXCR5+ HLA-DR+ B-cells could be observed in patients without cGVHD. As far as we are aware, this is a novel subset and most likely represents an activated B-cell population. It is unclear why patients without cGVHD would have an increased abundancy of this cluster and an increased frequency of CD38-expressing B-cells. As mentioned previously, CD38hi plasmablasts have been linked to cGVHD (58), however, the subset identified here remains putatively novel and warrants further investigation.

Differences in expression of cellular markers could also be observed between patients with moderate and severe cGVHD. While analyzing the flow cytometry panel for cluster 399948, we observed an increased frequency of CD39-expressing CXCR5+ HLA-DR+ B-cells in patients with severe cGVHD. One of the main functions of CD39 is to catalyze extracellular ATP, most commonly found in sites of tissue injury or death. Removing extracellular ATP has a dampening effect on the local immune system and as such CD39 is most often associated with regulatory T-cells (65). CD39 has hitherto not been associated with B-cells in the literature, not even with regulatory B-cells (66). Therefore, it is challenging to explain why patients with severe cGVHD have more activated B-cells expressing CD39 than patients with moderate cGVHD. To complicate matters more, if this subset is potentially a regulatory B-cell subset, previous studies have actually found regulatory B-cells to be decreased in cGVHD, not increased (58). It is possible that this could be an attempt by the immune system to dampen the activated B-cells, thought to be an important driver behind cGVHD pathogenesis. CD39 expression on B-cells is an interesting finding with little precedent which warrants further study.

Finally, patients with severe cGVHD had a reduced frequency of CD8-expressing CD57+ GzB+ T-cells compared to patients with moderate cGVHD. As discussed earlier, this specific subset could potentially be an NKT-cell subset or an activated cytotoxic T-cell subset. Lower frequencies of activated cytotoxic T-cells or NKT-cells in the blood of patients with severe cGVHD do not make much sense at first glance. However, it is possible that this cell population migrated from the blood and into the affected tissues, as discussed for the MAIT-cells. Analyzing patient biopsies could potentially elucidate this. Interestingly, purely looking at cytotoxic T-cells frequencies in these patients was not enough. The markers CD57 and GzB needed to be included to identify a specific subset of CD8+ T-cells that might play a role in severe cGVHD pathogenesis. To our knowledge, this subset has not been linked to cGVHD before. However, one recent study did identify another GzB positive subset linked to acute GVHD development (29). They assessed levels of GzB positive regulatory T-cells 30 days post-HSCT and found these to be present at elevated levels in patients with acute GVHD. As neither our mass cytometry nor flow cytometry panel included FoxP3, we were unable to assess any potential differences in GzB positive regulatory T cells. This could warrant further investigation.

Taken together, it would seem that patients with mild cGVHD display reduced frequencies of CD38-expressing activated B-cells and increased frequencies of activated cytotoxic T or NKT-cells compared to patients without cGVHD. Contrary to this, patients with severe cGVHD present with increased frequencies of CD39-expressing activated B-cells and reduced frequencies of activated cytotoxic T or NKT-cells compared to patients with moderate cGVHD. Differences in immunosuppressive regimens, which directly influences the immune phenotype extensively, might partially explain these contradictory results. This effect has been observed previously (23). It is crucial for future studies to take immunosuppressive regimens into account and, ideally, only include patients close to the time of cGVHD assessment when they have been exposed relatively shortly to the immunosuppressive drugs. Moreover, additional research in different cohorts is required to further elucidate these apparent incongruities and to determine if these markers may be used as potential diagnostic or predictive markers in the future. Validating the results in independent cohorts, preferably at a different center, is crucial before a marker may be considered to be used as a biomarker (32). In addition, in future studies performing a receiver operating characteristic (ROC) analysis to assess the prognostic potential of the markers would be vital, as has been done in many biomarker studies, both for acute and cGVHD (10, 11, 13, 14, 16, 25, 28, 29, 57). Unfortunately, the sample size in this study was insufficient to perform an ROC analysis correctly. Future studies should consider ample sample size to accommodate such an analysis.

It is encouraging that T-cell populations can first be identified using a high-dimensional analysis combined with unbiased clustering approach, and subsequently be monitored using a simpler flow cytometry panel, already available in clinical practice. This suggests a broader utility of such approaches in immune-monitoring of patients in general and patients undergoing immunotherapy in particular.

In this study, we confirm the difficulty in finding biomarkers for cGVHD in peripheral blood by means of standardized techniques looking at known cellular subsets. However, translating results from new high-dimensional mass cytometry techniques into feasible flow cytometry panels is possible and promising in the search for diagnostic and predictive cGVHD markers.

## Ethics Statement

This study was carried out in accordance with the recommendations of the Regional Ethical Review Board in Stockholm, Sweden (DNR 2007/1349-31 and DNR 2006/1433-31/3) with written informed consent from all subjects. All subjects gave written informed consent in accordance with the Declaration of Helsinki. The protocol was approved by the Regional Ethical Review Board in Stockholm, Sweden.

## Author Contributions

MU planned the study. AS, TL, ER, JG, and BS performed laboratory work. AB, MS, and JMa provided patient sample material. AS, YC, JT, and MR performed the data analysis. AS, PB, and MU interpreted the data. AS and MU wrote the manuscript. All coauthors critically revised the manuscript.

## Conflict of Interest Statement

The authors declare that the research was conducted in the absence of any commercial or financial relationships that could be construed as a potential conflict of interest.
