# Supplementary material for: Combining Flow and Mass Cytometry in the Search for Biomarkers in Chronic Graft-versus-Host Disease
Source: Front Immunol. 2017 Jun 19;8:717. doi: 10.3389/fimmu.2017.00717 (PMC5474470; doi:10.3389/fimmu.2017.00717)
Supplement: Supplementary file 1 [file Table_1.DOCX]

**Table S1. Methods used for each included patient.**

Seven patients were sampled at two different time points, indicated by “a” and “b”. cGVHD = Chronic Graft-Versus-Host-Disease; ELISA=enzyme-linked immunosorbent assay

| **Table S1. Methods** | | | | | | |
| --- | --- | --- | --- | --- | --- | --- |
| **cGVHD grade** | **Patient nr** | **Luminex** | **ELISA** | **Conventional flow cytometry** | **Mass cytometry** | **Confirmatory flow cytometry** |
| **none** | 1 | x | x | x | ... | ... |
|  | 2 | x | x | x | ... | ... |
|  | 3 | x | x | x | ... | ... |
|  | 4 | x | x | x | ... | ... |
|  | 5 | x | x | x | ... | ... |
|  | 6 | x | x | x | ... | ... |
|  | 7 | x | x | x | x | ... |
|  | 8 | x | x | x | x | ... |
|  | 9 | x | x | x | x | ... |
|  | 10 | x | x | x | x | ... |
|  | 11 | x | x | x | x | ... |
|  | 12 | ... | ... | ... | x | x |
|  | 13 | ... | ... | ... | x | x |
|  | 14 | ... | ... | ... | x | x |
|  | 15 | ... | ... | ... | x | x |
|  | 16 | ... | ... | ... | x | x |
|  | 17 | ... | ... | ... | x | x |
|  | 18a | ... | ... | ... | ... | x |
|  | 19 | ... | ... | ... | ... | x |
|  | 20 | ... | ... | ... | ... | x |
|  | 21 | ... | ... | ... | ... | x |
|  | 22 | ... | ... | ... | ... | x |
|  | 23 | ... | ... | ... | ... | x |
|  | 24 | ... | ... | ... | ... | x |
|  | 25 | ... | ... | ... | ... | x |
|  | 26 | ... | ... | ... | ... | x |
| **mild** | 18b | x | ... | ... | ... | ... |
|  | 27 | x | x | x | ... | ... |
|  | 28 | x | x | x | ... | ... |
|  | 29 | x | x | x | ... | ... |
|  | 30 | x | x | x | x | ... |
|  | 31 | x | x | x | x | ... |
|  | 32 | x | x | x | x | ... |
|  | 33 | x | ... | x | x | ... |
|  | 34 | ... | ... | ... | x | ... |
|  | 35 | ... | ... | ... | x | x |
|  | 36 | ... | ... | ... | x | x |
|  | 37 | ... | ... | ... | x | x |
|  | 38 | ... | ... | ... | x | x |
|  | 39 | ... | ... | ... | ... | x |
|  | 40 | ... | ... | ... | ... | x |
|  | 41a | ... | ... | ... | ... | x |
| **moderate** | 42 | x | x | x | ... | ... |
|  | 43 | x | x | x | x | ... |
|  | 44 | x | x | x | x | ... |
|  | 45 | x | x | x | x | ... |
|  | 41b | x | ... | x | x | ... |
|  | 46a | x | ... | x | ... | ... |
|  | 47 | x | ... | ... | x | ... |
|  | 48a | x | x | x | x | ... |
|  | 48b | ... | ... | ... | ... | x |
|  | 49a | x | ... | x | x | ... |
|  | 49b | ... | ... | ... | ... | x |
|  | 50 | x | ... | x | x | x |
|  | 51 | x | ... | ... | x | x |
|  | 52 | x | ... | ... | x | x |
|  | 53 | ... | ... | ... | ... | x |
|  | 54 | ... | ... | ... | ... | x |
|  | 55 | ... | ... | ... | ... | x |
|  | 56 | ... | ... | ... | ... | x |
|  | 57 | ... | ... | ... | ... | x |
| **severe** | 58 | x | x | x | x | ... |
|  | 59 | x | x | x | x | ... |
|  | 60 | x | x | x | x | ... |
|  | 61 | x | x | x | x | ... |
|  | 62 | x | x | x | x | ... |
|  | 63 | x | x | x | x | ... |
|  | 64 | x | ... | ... | x | ... |
|  | 65 | x | ... | x | ... | x |
|  | 66 | x | ... | ... | x | x |
|  | 46b | ... | ... | ... | ... | x |
|  | 67a | x | x | x | x | ... |
|  | 67b | ... | ... | ... | ... | x |
|  | 68a | x | x | x | x | ... |
|  | 68b | ... | ... | ... | ... | x |
