# Supplementary material for: Combining Flow and Mass Cytometry in the Search for Biomarkers in Chronic Graft-versus-Host Disease
Source: Front Immunol. 2017 Jun 19;8:717. doi: 10.3389/fimmu.2017.00717 (PMC5474470; doi:10.3389/fimmu.2017.00717)
Supplement: Supplementary file 2 [file Table_2.DOCX]

**Table S2. Flow cytometry antibodies.**

7-AAD=7-Amino-Actinomycin D; CCR=C-C chemokine receptor; CD=Cluster of differentiation; CTLA-4=cytotoxic T lymphocyte associated protein 4; CXCR=CXC chemokine receptor; Ig=Immunoglobulin; PD-1=programmed cell death protein 1; HLA-DR=human leukocyte antigen-antigen D related; TCR=T cell receptor; APC=allophycocyanin; PE=phycoerythrin; FITC=Fluorescein isothiocyanate; BD Biosciences, San Jose, CA, USA; Beckman Coulter, Immunotech, Marseille, France; BioLegend, San Diego, CA, USA; LifeSpan Biosciences, Seattle, WA, USA; R&D Systems Inc., Minneapolis, MN, USA

| **Table S2. Flow cytometry antibodies.** | | | |
| --- | --- | --- | --- |
| **Marker** | **Fluorochrome** | **Clone** | **Vendor** |
| 7-AAD | - | - | BD Biosciences |
| CCR9 | APC | #112509 | R&D Systems |
| CD3 | PE-Cy7 | SK7 | BD Biosciences |
|  | V450 | UCHT1 | BD Biosciences |
|  | BV510 | UCHT1 | BD Biosciences |
| CD4 | Alexa Fluor 700 | RPA-T4 | BD Biosciences |
|  | V500 | RPA-T4 | BD Biosciences |
|  | Krome Orange | 13B8.2 | Beckman Coulter |
| CD5 | APC | UCHT2 | BD Biosciences |
| CD8 | FITC | SK1 | BD Biosciences |
|  | V500 | RPA-T8 | BD Biosciences |
|  | APC-Cy7 | SK1 | BD Biosciences |
| CD11c | BV-421 | B-ly6 | BD Biosciences |
| CD16 | PE | 3G8 | BD Biosciences |
| CD19 | APC | HIB19 | BD Biosciences |
|  | Alexa Fluor 700 | HIB19 | BD Biosciences |
| CD20 | PE | L27 | BD Biosciences |
| CD25 | FITC | M-A251 | BD Biosciences |
| CD27 | V450 | M-T271 | BD Biosciences |
|  | PE | M-T271 | BD Biosciences |
|  | BV-421 | M-T271 | BD Biosciences |
| CD28 | PE-CF594 | CD28.2 | BD Biosciences |
|  | FITC | CD28.2 | BD Biosciences |
| CD38 | FITC | HIT2 | BD Biosciences |
| CD39 | BV-421 | TU66 | BD Biosciences |
|  | PE | TU66 | BD Biosciences |
| CD44 | APC | G44-26 | BD Biosciences |
| CD45RO | APC | UCHL1 | BD Biosciences |
| CD56 | FITC | NCAM16.2 | BD Biosciences |
|  | APC | B159 | BD Biosciences |
|  | Alexa Fluor 700 | B159 | BD Biosciences |
| CD57 | PE-CF594 | B3GAT1 | BD Biosciences |
| CD69 | FITC | L78 | BD Biosciences |
| CD94 | FITC | HP-3D9 | BD Biosciences |
| CD95 | FITC | DX2 | BD Biosciences |
| CD107a | FITC | H4A3 | BD Biosciences |
| CD127 | PE-Cy7 | HIL-7R-M21 | BD Biosciences |
|  | APC-Alexa Fluor 700 | R34.34 | Beckman Coulter |
| CD152/CTLA-4 γ | FITC | A3.4H2.H12 | LifeSpan Biosciences |
| CD158b | FITC | CH-L | BD Biosciences |
| CD158b KIR2DL2/L3 NKAT2 | PE-Cy7 | DX27 | Biolegend |
| CD161 | PE | HP-3G10 | Biolegend |
| CD183/CXCR3 | APC | IC6/CXCR3 | BD Biosciences |
| CD185/CXCR5 | BV510 | RF8B2 | BD Biosciences |
| CD194/CCR4 | PE | 1G1 | BD Biosciences |
| CD196/CCR6 | FITC | G034E3 | Biolegend |
| CD197/CCR7 | PE-Cy7 | 3D12 | BD Biosciences |
| CD279/PD-1 | BV-421 | MIH4 | BD Biosciences |
| Fixable Viability Stain 780 | - | - | BD Biosciences |
| Granzyme B | Alexa Fluor 700 | GB11 | BD Biosciences |
| HLA-DR | PE-CF594 | G46-6 | BD Biosciences |
| IgD | FITC | IA6-2 | BD Biosciences |
| IgM | FITC | G20-127 | BD Biosciences |
| Ki-67 | PE-Cy7 | B56 | BD Biosciences |
| PD-1 | FITC | MIH4 | BD Biosciences |
| TCR PANγδ | FITC | IMMU510 | Beckman Coulter |
| TCR αβ | FITC | WT31 | BD Biosciences |
| TCRVα7.2 | APC-Cy7 | 3C10 | Biolegend |
