# Supplementary material for: Combining Flow and Mass Cytometry in the Search for Biomarkers in Chronic Graft-versus-Host Disease
Source: Front Immunol. 2017 Jun 19;8:717. doi: 10.3389/fimmu.2017.00717 (PMC5474470; doi:10.3389/fimmu.2017.00717)
Supplement: Supplementary file 3 [file Table_3.DOCX]

**Table S3. Mass cytometry antibodies.**

CD=Cluster of differentiation; CTLA-4=cytotoxic T lymphocyte associated protein 4; CCR=C-C chemokine receptor; ICOS=inducible costimulatory molecule; PD-1=programmed cell death protein 1; CXCR=CXC chemokine receptor; TCR=T cell receptor; HLA-DR=human leukocyte antigen-antigen D related; Sm=Samarium; Nd=Neodymium; Er=Erbium; Gd=Gadolinium; Yb=Ytterbium; Dy=Dysprosium; Y=Yttrium; Tm=Thulium; In=Indium; Ho=Holmium; Gd=Gadolinium; Pr=Praseodymium; Eu=Europium; Pt=Platinum; Ir=Iridium; Fluidigm, CA, USA; BioLegend, San Diego, CA, USA; R&D Systems Inc., Minneapolis, MN, USA

| **Table S3. Mass cytometry antibodies.** | | | |
| --- | --- | --- | --- |
| **Marker** | **Tag** | **Clone** | **Vendor** |
| CD3ε | 154Sm | UCHT1 | Fluidigm |
| CD4 | 145Nd | RPA-T4 | Fluidigm |
| CD5 | 143Nd | UCHT2 | BioLegend |
| CD8α | 146Nd | SK1 | BioLegend |
| CD11c | 147Sm | Bu15 | Fluidigm |
| CD19 | 142Nd | HIB19 | Fluidigm |
| CD27 | 167Er | L128 | Fluidigm |
| CD28 | 160Gd | CD28.2 | BioLegend |
| CD31 | 148Nd | WM59 | BioLegend |
| CD38 | 168Er | HIT2 | BioLegend |
| CD39 | 173Yb | A1 | BioLegend |
| CD44 | 164Dy | BJ18 | BioLegend |
| CD45 | 89Y | HI30 | Fluidigm |
| CD45RA | 169Tm | HI100 | Fluidigm |
| CD57 | 115In | HCD57 | BioLegend |
| CD127 | 165Ho | A019D5 | Fluidigm |
| CD152/CTLA-4 | 170Er | 14D3 | Fluidigm |
| CD161 | 161Dy | HP-3G10 | BioLegend |
| CD194/CCR4 | 155Gd | 205410 | R&D Systems |
| CD195/CCR5 | 144Nd | NP-6G4 | Fluidigm |
| CD196/CCR6 | 141Pr | G034E3/11A9 | BioLegend/Fluidigm |
| CD278/ICOS | 151Eu | DX29 | Fluidigm |
| CD279/PD-1 | 172Yb | EH12.2H7 | BioLegend |
| CXCR3 | 157Gd | G025H7 | BioLegend |
| CXCR5 | 174Yb | 51505 | R&D Systems |
| TCRγδ | 152Sm | 11F2 | Fluidigm |
| TCRαβ | 152Sm | IP26 | BioLegend |
| HLA-DR | 163Dy | L243 | BioLegend |
| Ki-67 | 162Dy | B56 | Fluidigm |
| Granzyme B | 171Yb | GB11 | Fluidigm |
| Cell-ID™ Cisplatin (Live-dead) | 195Pt | - | Fluidigm |
| Cell-ID™ Intercalator-Ir (DNA) | 191Ir | - | Fluidigm |
| Cell-ID™ Intercalator-Ir (DNA) | 193Ir | - | Fluidigm |
