# Supplementary material for: Combining Flow and Mass Cytometry in the Search for Biomarkers in Chronic Graft-versus-Host Disease
Source: Front Immunol. 2017 Jun 19;8:717. doi: 10.3389/fimmu.2017.00717 (PMC5474470; doi:10.3389/fimmu.2017.00717)
Supplement: Supplementary file 4 [file Table_4.DOCX]

**Table S4. Flow cytometry marker staining panels.**

CD=Cluster of differentiation; TCR=T cell receptor; 7-AAD=7-Amino-Actinomycin D; CCR=C-C chemokine receptor; CTLA-4=cytotoxic T lymphocyte associated protein 4; CXCR=CXC chemokine receptor; Ig=Immunoglobulin; PD-1=programmed cell death protein 1; HLA-DR=human leukocyte antigen-antigen D related;

| **Table S4. Flow cytometry marker staining panels.** | |
| --- | --- |
| **Conventional flow panel** | |
| T cell panel | CD3, CD4, CD8, CCR7, CD45RO, CD27, CD127, CD69, CD94, CD56, CD95, PD-1, CD107a, CD38, CTLA-4, CD158b, CD28, TCRαβ, TCRγδ, 7-AAD |
| Treg cell panel | CD3, CD4, CD8, CCR7, CD45RO, CD25, CD39, CD127, 7-AAD |
| MAIT cell panel | CD3, CD4, CD8, CD161, TCRVα7.2, CCR6, CCR9, 7-AAD |
| B cell panel | CD3, CD56, CD19, CD20, CD27, IgM, IgD, CD25, CD38, PD-1, 7-AAD |
| NK cell panel | CD3, CD4, CD8, CD56, CD16, CD94, CD158b, CD27, 7-AAD |
|  |  |
| **Confirmatory flow panel** | |
| B cell panel | CD19, HLA-DR, CD38, CD39, CXCR3, CXCR5, CCR4, Ki-67, viability |
| NKT cell panel | CD3, CD8, CD57, Granzyme B, CCR4, CD44, PD-1, Ki-67, viability |
| NK cell panel | CD3, CD56, Granzyme B, CD57, CD161, CD38, CD11c, Ki-67, viability |
| CD4 T cell panel | CD3, CD4, CD5, CD27, CD28, TCRαβ, CCR4, CD127, viability |
