# Supplementary figures and images for: Combining Flow and Mass Cytometry in the Search for Biomarkers in Chronic Graft-versus-Host Disease
Source: Front Immunol. 2017 Jun 19;8:717. doi: 10.3389/fimmu.2017.00717 (PMC5474470; doi:10.3389/fimmu.2017.00717)

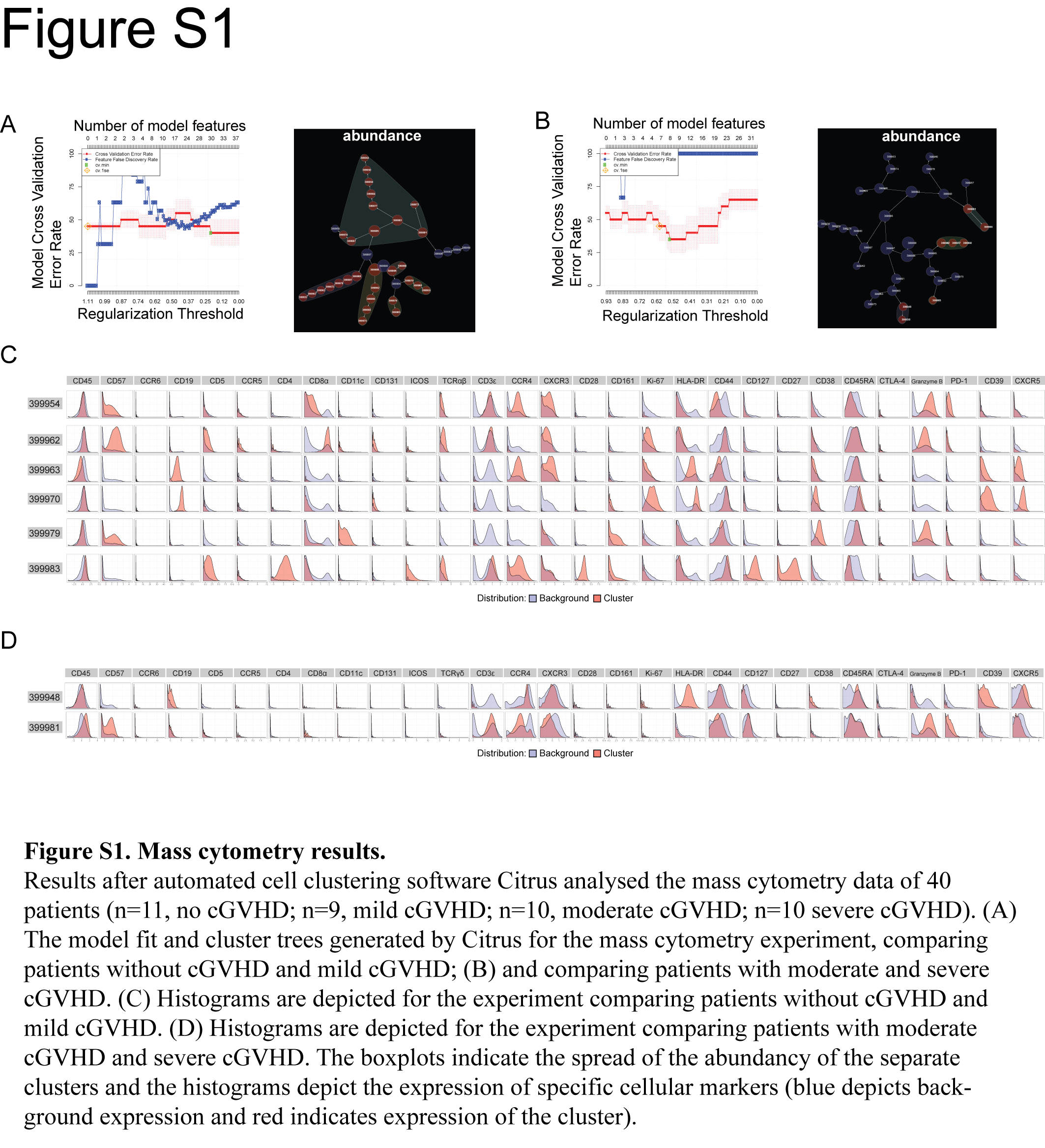

Supplement: Supplementary file 5 [file Image_1.TIF]

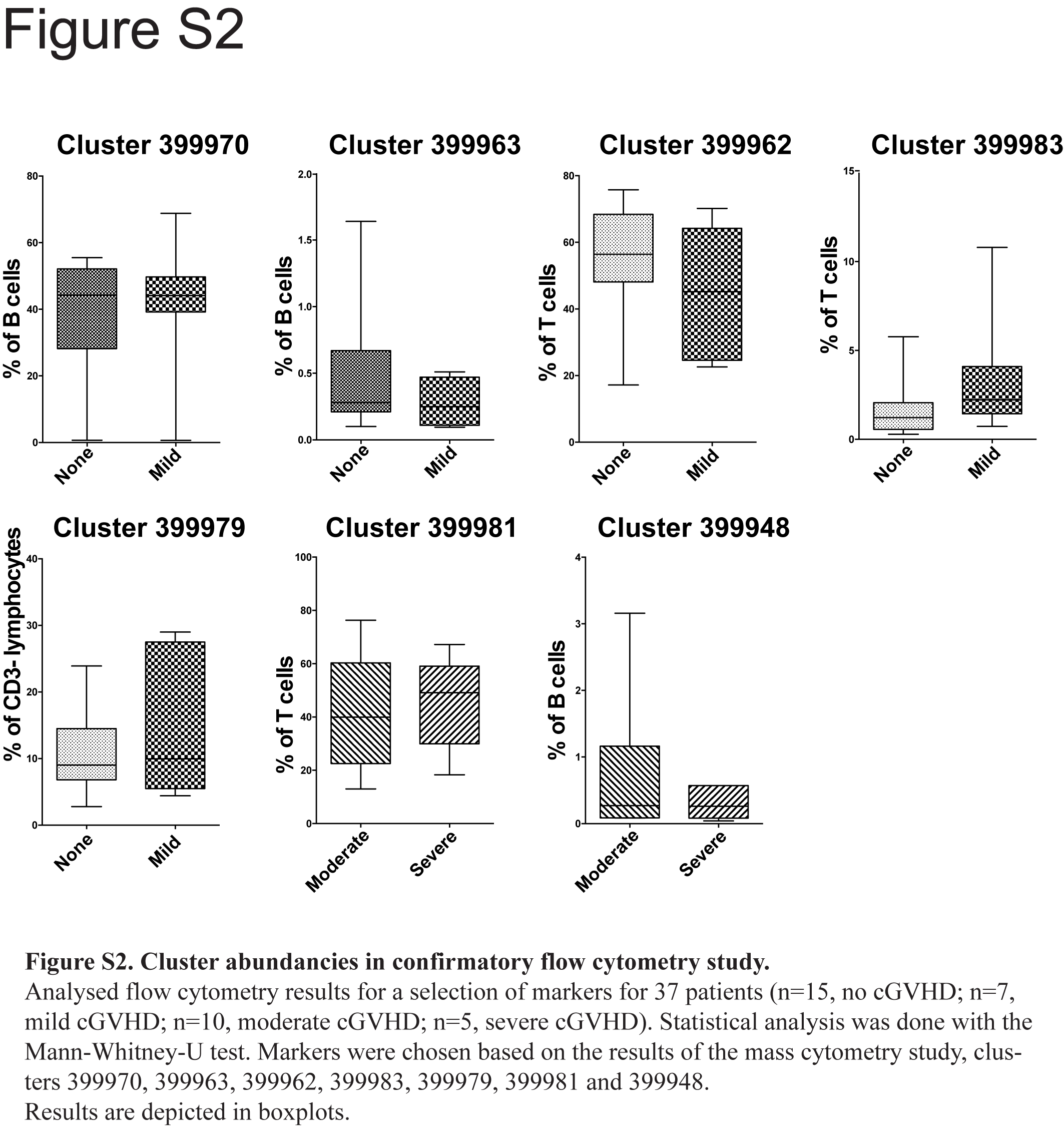

Supplement: Supplementary file 6 [file Image_2.TIF]
